# Anastomotic Dehiscence Secondary to Early Eventration in Abdominal Trauma Caused by a Bull Horn: The Critical Importance of Abdominal Wall Integrity

**DOI:** 10.7759/cureus.106035

**Published:** 2026-03-28

**Authors:** Christopher Kaleb Romero Ríos, Nilson Ortiz, Sergio Dávila, Christian Sequeira, Carlos Romero Manfut

**Affiliations:** 1 School of Medicine, Hospital Militar Escuela "Dr. Alejandro Dávila Bolaños", Managua, NIC; 2 General Surgery, Hospital Militar Escuela "Dr. Alejandro Dávila Bolaños", Managua, NIC; 3 Urology, Hospital Militar Escuela "Dr. Alejandro Dávila Bolaños", Managua, NIC

**Keywords:** abdominal trauma, anastomotic dehiscence, bull horn injury, damage control surgery, eventration

## Abstract

Abdominal trauma caused by a bull horn is a rare but severe injury that can lead to multiple intestinal perforations and life-threatening complications. This case report highlights the critical management challenges and emphasizes the importance of multidisciplinary care in a high-risk surgical patient. A 36-year-old male presented with open abdominal trauma and small bowel evisceration following a bull horn injury. An initial exploratory laparotomy revealed three perforations in the ileum, requiring a 30 cm resection and end-to-end anastomosis. Postoperatively, he was managed in the intensive care unit due to the severity of the trauma. On postoperative day two, he developed signs of intestinal obstruction and underwent a second exploratory laparotomy. Intraoperative findings revealed ileal eventration through the previous penetrating wound, resulting in partial dehiscence of a single suture at the mesenteric border of the anastomosis. To our knowledge, this is one of the few documented cases in which early eventration directly led to anastomotic dehiscence following bull horn trauma. The anastomosis was reconstructed, and the abdominal wall was reinforced. The postoperative course was complicated by acute kidney injury, which improved with fluid resuscitation and withdrawal of nephrotoxic agents. The patient was discharged on postoperative day twelve with oral antibiotics and scheduled outpatient follow-up. This case illustrates a rare but severe complication following high-energy penetrating trauma and underscores the critical importance of considering damage control surgery in cases with extensive contamination. The favorable outcome highlights that maintaining a high index of suspicion for complications, early re-intervention, meticulous surgical technique, and coordinated multidisciplinary management are key to successful outcomes in complex abdominal trauma.

## Introduction

Penetrating abdominal trauma caused by a bull horn is uncommon worldwide but remains clinically relevant in regions with bull-related festivities. These injuries are characterized by irregular trajectories and high-impact energy capable of producing complex visceral and vascular damage [1]. In a 40-year retrospective series from Spain, 572 hospital admissions due to bull horn injuries were reported, with the abdomen among the most commonly affected sites [2]. A multicenter study conducted in Spain, Portugal, and France reported low mortality (0.48%) but significant morbidity, particularly in abdominal or vascular trauma [3]. Frequent intestinal eventration increases the risk of postoperative complications such as anastomotic leakage, infections, and sepsis, requiring prompt surgical intervention and structured postoperative care. For clarity, “eventration” refers to the protrusion of abdominal contents through a defect in the abdominal wall, whereas “anastomotic dehiscence” denotes the partial or complete breakdown of a surgically created intestinal connection. This report aims to illustrate how early abdominal wall failure can precipitate anastomotic dehiscence and to discuss preventive strategies that may reduce postoperative complications. We report the case of a patient with severe abdominal injury due to a bull horn and iatrogenic ileal anastomotic dehiscence, emphasizing surgical challenges and the importance of vigilant postoperative follow-up.

## Case presentation

**Figure 1:**
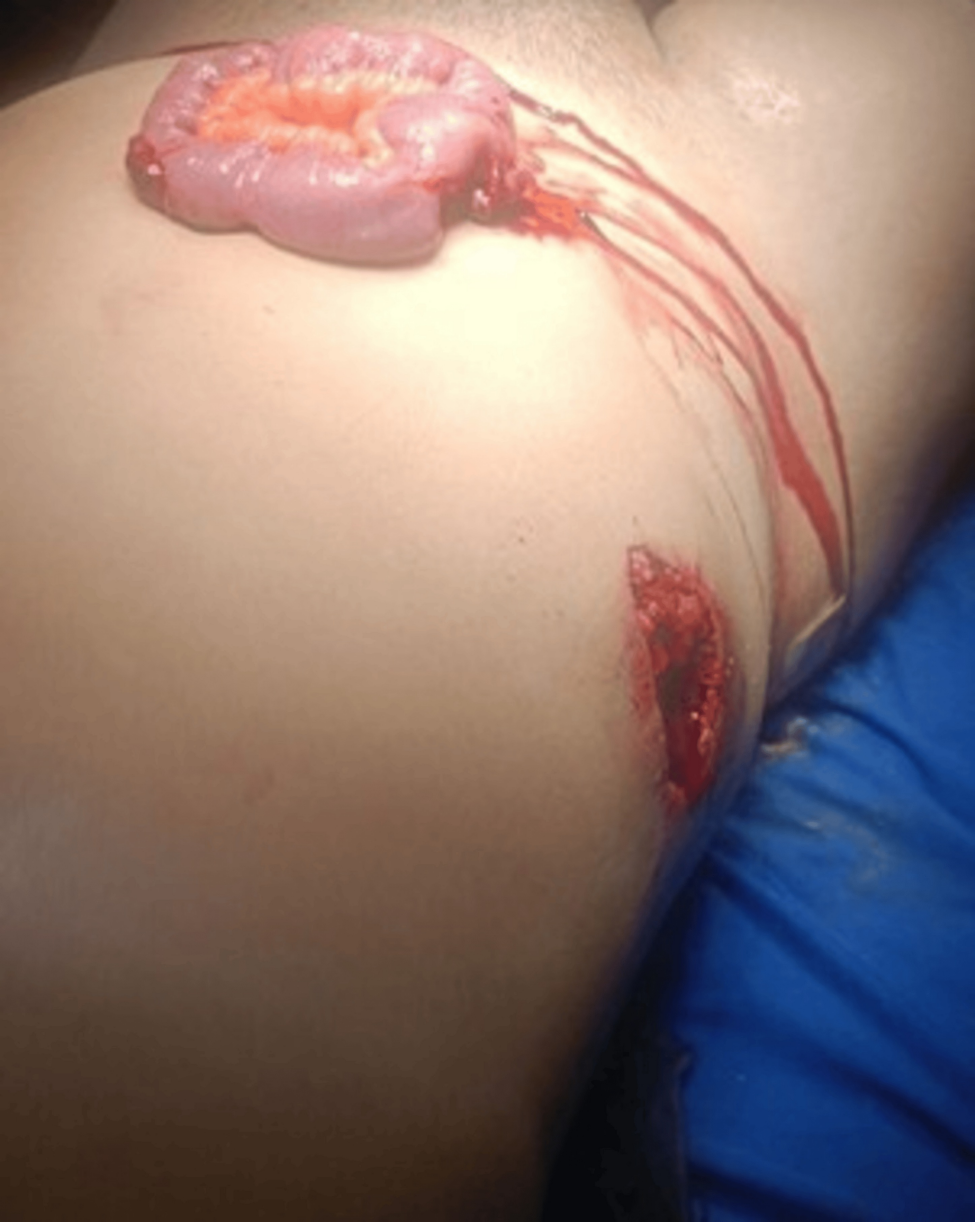
View of the two penetrating abdominal wounds. Evisceration of a small bowel loop is observed through the 8 cm right iliac fossa wound, and a second 10 cm bleeding wound is present in the right flank, both consistent with a bull’s horn injury.

A 36-year-old previously healthy male presented to the emergency department following an open abdominal injury from a bull horn. Initial evaluation revealed two penetrating wounds: one approximately 10 cm in length in the right flank and another 8 cm in the right iliac fossa with small bowel evisceration (Figure 1). Upon arrival, vital signs were stable: blood pressure 120/74 mmHg, heart rate 78 bpm, respiratory rate 18 breaths per minute, and oxygen saturation 98%. Laboratory tests showed marked leukocytosis of 22.50×10³/μL with 87.5% neutrophils, indicating a significant inflammatory response.

**Figure 2:**
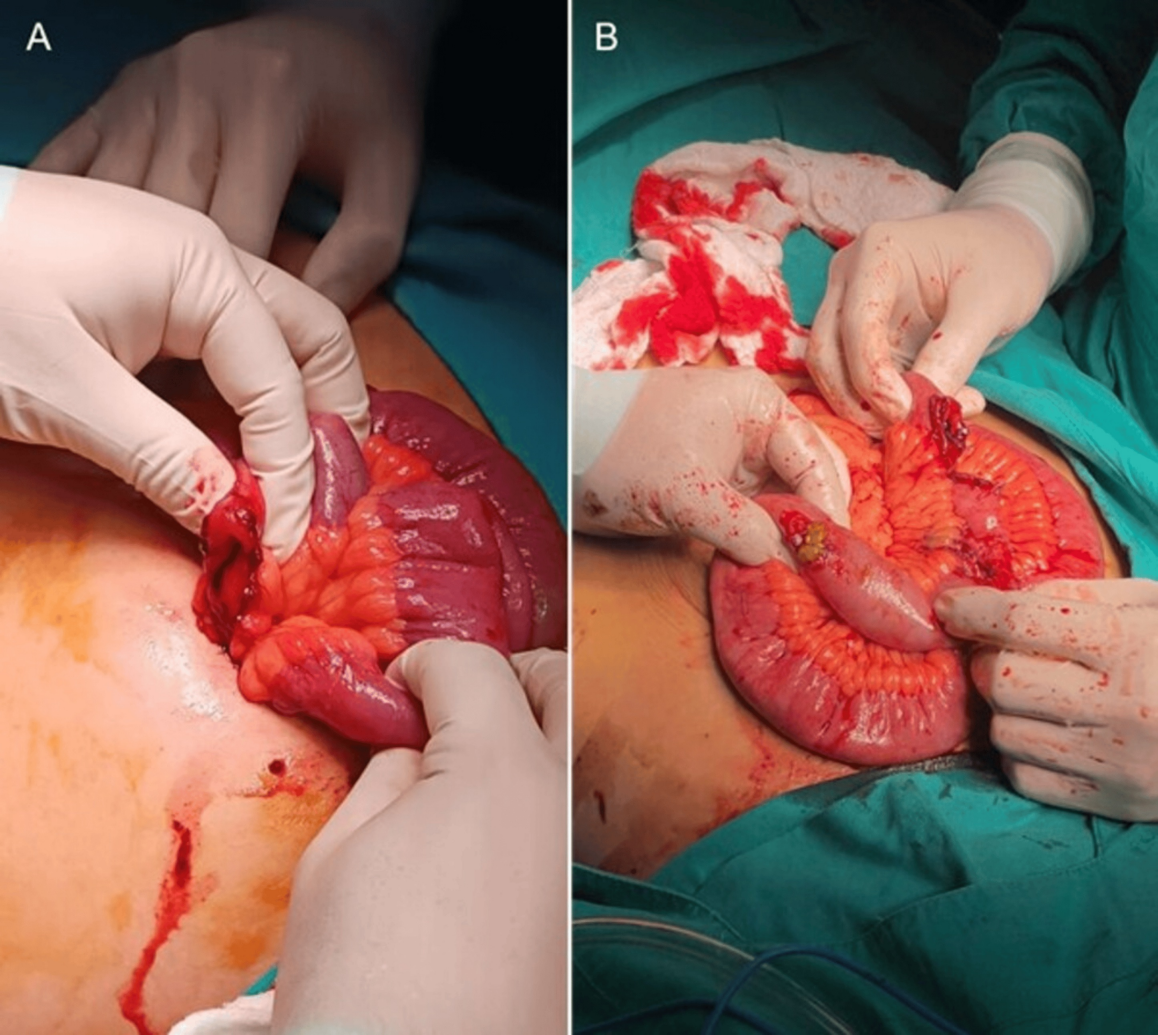
Intraoperative views showing multiple ileal perforations with active bleeding (A) A close-up view of the three perforations with intestinal leakage (B), indicating the need for resection due to the extent of the injury.

Given the severity of the injuries, the patient was immediately taken to the operating room for emergency exploratory laparotomy. The right iliac wound was extended to a supraumbilical midline incision. Three perforations in the ileum were identified, located 200 cm from the ligament of Treitz and 110 cm from the ileocecal valve. Approximately 200 mL of intestinal-fecal content and 400 mL of blood were present in the peritoneal cavity. No other organ injuries were found. A 30 cm segment of the ileum was resected, and an end-to-end anastomosis was performed (Figure 2). The anastomosis was constructed using a continuous 3-0 polydioxanone suture in a Connell-Mayo pattern and reinforced with seromuscular Lembert sutures using 3-0 silk. The mesenteric defect was closed with 3-0 polyglactin suture. The abdominal cavity was irrigated with 5000 mL of warm saline, and two Jackson-Pratt drains were placed. The abdominal wall was closed in layers: fascia and rectus muscles with 1-0 Polyglactin suture, subcutaneous tissue with 2-0 polyglactin suture, and skin with 3-0 nylon.

**Figure 3:**
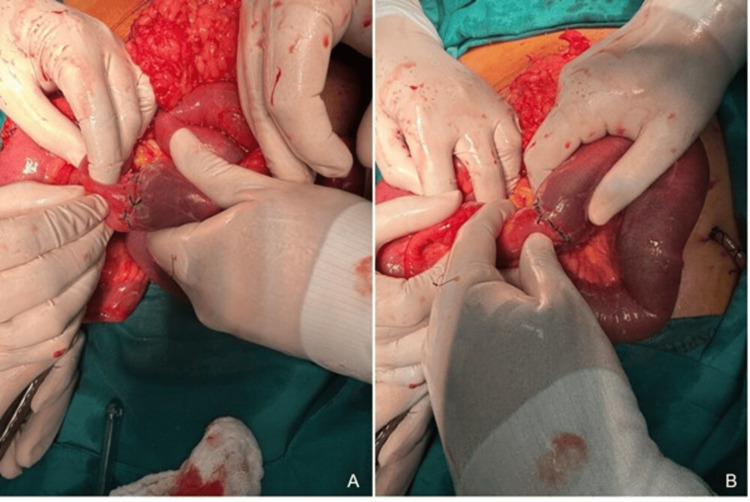
Intraoperative images of the reoperation. (A) Ileal loop with reconstructed end-to-end anastomosis after eventration repair. (B) Close-up view showing the corrected dehiscence and viable tissue after surgical revision.

At 48 hours post-surgery, the patient developed abdominal distension and pain, though vital signs remained stable. A re-exploration was performed, revealing eventration of an ileal loop specifically through the disruption of the original penetrating wound. It is important to note that the fascial closure of the surgical midline incision remained intact. This caused partial anastomotic dehiscence at the mesenteric border, likely due to tension from intestinal distension (Figure 3). The abdominal wall defect was repaired by approximating the oblique muscles and closing the fascia with 1-0 polyglactin suture. The subcutaneous layer was closed with a purse-string 1-0 polyglactin suture, and a povidone-iodine gauze dressing was applied. Peritoneal lavage and subcutaneous Jackson-Pratt drainage were performed. Broad-spectrum antibiotics, piperacillin-tazobactam (4.5 g every 6 hours) and metronidazole (500 mg every 8 hours), were initiated. The patient was monitored in the intensive care unit (ICU).

During his ICU stay, he developed acute kidney injury (AKI) with a peak creatinine of 3.75 mg/dL and blood urea nitrogen (BUN) of 53 mg/dL on postoperative day 4. The nephrology team attributed this to a prerenal component aggravated by rhabdomyolysis secondary to trauma. Fluid therapy was optimized, and nephrotoxic agents were withdrawn. The patient also experienced paralytic ileus, managed conservatively with nasogastric decompression and specialized enteral nutrition.

By postoperative day 10, the patient tolerated a liquid diet, the drain output decreased, and his overall condition stabilized. He was discharged on day 12 with improving labs (hematocrit 33.3%, leukocytes 12.32×10³/μL). The surgical wounds were healing adequately. He was instructed on wound care, physical activity restriction, and warning signs. Oral amoxicillin-clavulanic acid plus metronidazole was prescribed for 10 days, and an outpatient follow-up visit was scheduled for suture removal.

## Discussion

Penetrating abdominal trauma due to a bull horn is a rare clinical entity but holds significance in countries with bullfighting traditions such as Spain, Mexico, and parts of Latin America, although the discontinuation of these practices could serve as an important preventive measure [1,3]. Unlike other penetrating injuries, bull horn trauma combines low-velocity but high-energy impact, unpredictable trajectories, and high bacterial contamination from the animal’s flora and the patient’s clothing [3]. This triad results in extensive tissue injury and frequent intestinal perforations with evisceration, leading to high postoperative morbidity despite relatively low mortality [2-4].

Anastomotic dehiscence remains one of the most feared complications, with an incidence ranging from 1% to 10% in trauma settings, depending on injury severity and risk factors [5,6]. In this case, the complication provided valuable lessons beyond technical failure. Management of such patients illustrates the complexity of severe abdominal trauma, where success depends not only on surgical skill but also on anticipating mechanical and physiological insults.

Performing a primary end-to-end anastomosis was consistent with current trends favoring primary repair in hemodynamically stable patients with penetrating bowel injuries [7-10]. However, this case lay at the threshold of that recommendation. Massive fecal contamination, significant hemorrhage, and the high-energy nature of the wound are established risk factors for anastomotic failure [6]. Under such conditions, a damage control surgery (DCS) strategy-rapid resection, bowel ends ligation, temporary abdominal closure, and planned re-look laparotomy 24-48 hours later-would have been a prudent alternative [9]. This approach limits initial operative time and avoids creating an anastomosis in a physiologically unstable patient with visceral edema.

The mechanism of dehiscence in this patient was mechanical rather than ischemic or infectious: early eventration through the traumatic wall defect imposed excessive tension on the suture line. This underscores a key principle in penetrating trauma: the abdominal wall sustains a “triple insult”: (1) direct tissue destruction, (2) surgical incision, and (3) postoperative intra-abdominal pressure elevation. Together, these render the traumatic wound the point of least resistance. Literature on “open abdomen” management suggests that in cases of extensive contamination or parietal tissue loss, reinforcement with mesh or fascial tension-mediated closure techniques may be necessary to prevent acute eventration [11-14]. Failure to secure the “container” (the abdominal wall) directly led to failure of the “content” (the anastomosis).

Additionally, the development of AKI due to rhabdomyolysis illustrates that severe trauma is a systemic disease. Post-traumatic AKI is an independent predictor of mortality [13]. In this case, multifactorial causes-direct muscle injury, transient hypoperfusion, and inflammatory mediator release exacerbated by sepsis-were identified. Recovery depended on multidisciplinary management in the ICU, including nephrology consultation for fluid optimization and nephrotoxin avoidance.

The patient’s favorable outcome, with discharge free of major sequelae, reflects effective coordination between surgery, critical care, and nutrition services, along with prophylactic antibiotics, meticulous wound care, and close sepsis surveillance. This case reinforces the importance of maintaining a high index of suspicion for early complications and prompt surgical re-intervention, ensuring comprehensive management in complex abdominal trauma. A major learning point from this case is the critical role of early recognition and support of the abdominal wall after high-energy penetrating trauma, as failure in this regard directly contributed to anastomotic dehiscence. Optimizing abdominal wall integrity through timely reinforcement or temporary closure techniques may prevent such postoperative complications.

## Conclusions

The management of this penetrating abdominal trauma complicated by anastomotic dehiscence secondary to early eventration underscores the importance of an integrative surgical strategy guided by damage control principles. Although primary closure and anastomosis were initially successful, subsequent abdominal wall failure highlights a critical lesson consistent with international guidelines: in high-energy trauma with evisceration and contamination, the integrity of the abdominal wall closure is as vital as visceral repair itself. Current evidence suggests that in high-risk scenarios such as this, DCS or delayed closure should be strongly considered to reduce postoperative complications. Ultimately, success depends on risk anticipation, early reoperation when indicated, and coordinated multidisciplinary care ensuring both mechanical and physiological stability in the polytrauma patient. As a practical recommendation, surgeons should consider reinforcing traumatic abdominal wall defects during the initial surgery to prevent early eventration and related anastomotic failure.
